# The association of depression and diabetes across methods, measures, and study contexts

**DOI:** 10.1186/s40842-017-0052-1

**Published:** 2018-01-04

**Authors:** Jaimie C. Hunter, Brenda M. DeVellis, Joanne M. Jordan, M. Sue Kirkman, Laura A. Linnan, Christine Rini, Edwin B. Fisher

**Affiliations:** 1 Department of Social Sciences and Health Policy, Division of Public Health Sciences, Wake Forest University School of Medicine, Medical Center Boulevard, Winston-Salem, NC 27157 USA; 2 Department of Health Behavior, Gillings School of Global Public Health, The University of North Carolina at Chapel Hill, Chapel Hill, NC USA; 3 Division of Rheumatology, Allergy, and Immunology, Thurston Arthritis Research Center, University of North Carolina School of Medicine, Chapel Hill, NC USA; 4 Diabetes Care Center Clinical Trials Unit, Division of Endocrinology and Metabolism, University of North Carolina School of Medicine, Chapel Hill, NC USA; 5 Cancer Prevention and Control, John Theurer Cancer Center, Hackensack University Medical Center, Hackensack, NJ USA; 6 Department of Biomedical Research, Hackensack University Medical Center, Hackensack, NJ USA; 7 Department of Oncology, Georgetown University School of Medicine, Washington DC, USA

**Keywords:** Depression, Diabetes, Psychosocial, Mental health

## Abstract

**Background:**

Empirical research has revealed a positive relationship between type 2 diabetes mellitus and depression, but questions remain regarding timing of depression measurement, types of instruments used to measure depression, and whether “depression” is defined as clinical depression or depressive symptoms. The present study sought to establish the robustness of the depression-diabetes relationship across depression definition, severity of depressive symptoms, recent depression, and lifetime depression in a nationally representative dataset and a large rural dataset.

**Methods:**

The present examination, conducted between 2014 and 2015, used two large secondary datasets: the National Health and Nutrition Examination Survey (NHANES) from 2007 to 2008 (*n* = 3072) and the Arthritis, Coping, and Emotion Study (ACES) from 2002 to 2006 (*n* = 2300). Depressive symptoms in NHANES were measured using the Patient Health Questionnaire 9-item survey (PHQ-9). ACES used the Center for Epidemiologic Studies—Depression Scale (CES-D) to measure depressive symptoms and the Composite International Diagnostic Interview (CIDI) to measure diagnosable depression. Diabetes was modelled as the dichotomous outcome variable (presence vs. absence of diabetes). Logistic regression was used for all analyses, most of which were cross-sectional. Analyses controlled for age, ethnicity, sex, education, and body mass index, and NHANES analyses used sample weights to account for the complex survey design. Additional analyses using NHANES data focused on the addition of health behavior variables and inflammation to the model.

**Results:**

*NHANES.* Every one-point increase in depressive symptoms was associated with a 5% increase in odds of having diabetes [OR: 1.05 (CI: 1.03, 1.07)]. These findings persisted after controlling for health behaviors and inflammation. *ACES*. For every one-point increase in depressive symptom score, odds of having diabetes increased by 2% [OR: 1.02 (CI: 1.01, 1.03)]. Recent (past 12 months) depression [OR: 1.49, (CI: 1.03, 2.13)] and lifetime depression [OR: 1.40 (CI: 1.09, 1.81)] were also significantly associated with having diabetes.

**Conclusions:**

This study provides evidence for the robustness of the relationship between depression or depressive symptoms and diabetes and demonstrates that depression occurring over the lifetime can be associated with diabetes just as robustly as that which occurs more proximal to the time of study measurement.

## Background

Depression is devastating to productivity, relationships, and overall well-being and, in the United States (US), remains second only to back pain as a leading cause of disability [1]. Despite having an estimated lifetime prevalence of 20% in adults [2], it frequently goes undiagnosed and untreated, owing in part to a dramatic shortage of mental health care providers [3]. Untreated depression is associated with greater quantity and severity of physical and mental health comorbidities [4]. For instance, depression is frequently comorbid with sleep problems, [5] anxiety, [6] and cardiovascular disease [7]. Depression is often associated with risky health behaviors, such as poor diet, sedentariness, and smoking, which, in turn, increase risk for chronic diseases like obesity and diabetes [8].

Diabetes mellitus, a frequent comorbidity, impacts 26 million Americans. Approximately 95% of cases are type 2 [9]. The prevalence of diabetes has increased rapidly, fueled by a worldwide increase in obesity, population aging, and longer life expectancies [10]. Altering behavioral risk factors like unhealthy diet and sedentariness reduces diabetes risk [11, 12]. Innovative prevention efforts are needed, and, therefore, interest in psychosocial approaches to preventing diabetes has grown in recent years [13–15]. As one example, peer support programs, in which trained helpers offer guidance and support for people at risk for a health condition, have proven invaluable in encouraging individuals to follow recommended health behaviors. Improved health behaviors, in turn, reduce risk for diabetes and other chronic diseases [16, 17].

People with depression are more likely to have diabetes than those in the general population [18]. The reverse is also true; depression prevalence among individuals living with diabetes is estimated to range from 9% to 35% [19, 20]. Bidirectional pathways drive this comorbidity. Researchers using a decade of data from the Nurses’ Health Study found a 17% increase in diabetes risk for individuals with depression and a 29% increase in depression risk for those with diabetes [21]. Another study showed a 43% increase in depression risk over six years for participants with baseline diabetes and a 102% increase in risk for diabetes among those with depression at baseline [22].

Knowing whether timing of depression diagnosis (recent versus at any point over the lifetime) matters in association with diabetes risk would prove informative for preventive interventions. The goal of the present study was therefore to explore the robustness of the relationship between depressive symptoms and diabetes status across varied samples, measures, and contexts. Analyses for this study were based on two distinct samples: a large, nationally representative sample (the National Health and Nutrition Examination Survey [NHANES]) [23] and a population sample addressing arthritis and other chronic diseases among rural communities in North Carolina (the Arthritis, Coping, and Emotion Study [ACES]), part of a larger study of osteoarthritis in the region known as the Johnston County Osteoarthritis Project (JoCo OA) [24]. Researchers hypothesized a robust, positive relationship between depression and diabetes status.

## Methods

### Study samples

The present investigation, conducted between 2014 and 2016, was a secondary analysis of data from two independent studies: NHANES and ACES. NHANES is a continuing series of independent studies of chronic disease risk factors among non-institutionalized civilians in the United States. Conducted in person, the study draws data from interviews and laboratory measures. The de-identified data are freely available from the National Center for Health Statistics [23]. Because the ACES study enrolled only individuals 45 years of age and older, the present NHANES analyses were restricted to adults in this age range.

ACES data were collected between 2002 and 2006 from a subset of individuals who participated in JoCo OA, a longitudinal, epidemiological study of knee and hip osteoarthritis in rural North Carolina (NC) [24]. Using in-person, in-depth interviews, ACES investigators collected data to identify psychosocial, behavioral, and disease-related factors that mitigate or exacerbate psychiatric comorbidity with physical illness in older adults. ACES included self-reported psychosocial data and collected participants’ weight and height. Inclusion criteria were being Caucasian or African American, living in Johnston County, being a civilian, and being at least 45 years of age. The first wave of data, collected from 2001 to 2006, was used in the present study.

### Measures

#### Depression

NHANES included the nine item Patient Health Questionnaire (PHQ-9) [25, 26]. Items assess the frequency of depressive symptoms as categorized in the fourth edition of the *Diagnostic and Statistical Manual for Mental Disorders* (DSM-IV) (e.g., “little interest or pleasure in doing things”), and responses are made on the following scale: not at all (0), several days (1), more than half the days (2), and nearly every day (3). Responses were summed to create a score with a possible range of 0 to 27; higher scores indicate higher levels of depressive symptoms. The PHQ-9 has been validated in individuals living with diabetes [27].

ACES included the Composite International Diagnostic Interview (CIDI), [28] which can identify probable cases of depression that occurred over the lifetime and over the previous 12 months using an algorithm based on third edition of the *Diagnostic and Statistical Manual for Mental Disorders, Revised* (DSM-III-R) criteria. The study also assessed depressive symptoms using the Center for Epidemiologic Studies–Depression (CES-D) scale [29]. The CES-D contains 20 items (e.g., “I was bothered by things that usually don't bother me”), and participants rated how often they were true: rarely or none of the time (0); some or a little of the time (1); occasionally or a moderate amount of time (2); or most or all of the time (3). The CES-D responses were summed to create a score of 0 to 60, with higher scores indicating higher levels of depressive symptoms. Reverse coding was used where appropriate.

### Diabetes

Type 2 diabetes was a dichotomous outcome variable in both studies. The 2007–2008 NHANES study followed standards outlined by the Centers for Disease Control and Prevention (CDC): a respondent was classified as having diabetes if he or she had a fasting plasma glucose (FPG) level of at least 126 or a glycated hemoglobin (HbA_1_c) level of at least 6.5, or if he or she reported having a doctor’s diagnosis of diabetes [9]. For ACES, diabetes was defined using a dichotomous, self-reported item indicating that a doctor had “ever told” the respondent that he or she has diabetes or high blood sugar. Recent studies examining the veracity of self-reported diabetes status as compared to health insurance administrative records and measured diabetes indices revealed very strong correlations between the measures [30, 31].

### Covariates

Both studies controlled for sex (female = 1, male = 0); ethnicity (Caucasians = 0, African Americans = 1, NHANES Latino = 2, NHANES Other = 3); age; body mass index (BMI); and education (high school or less = 1, more than high school education = 0). Income data were not collected in the ACES study because investigators felt they were too sensitive.

### Statistical analysis

Analyses were conducted using SAS version 9.3 software (SAS Institute, Cary, NC). Descriptive statistics were used to examine the distribution of all variables. Univariate analyses of quantitative (continuous) variables proceeded using PROC MEANS and PROC UNIVARIATE to determine range, skewness and kurtosis, and statistical mean and standard deviation. PROC FREQ was used to provide a descriptive examination of each categorical variable. It was unnecessary to transform any variable to facilitate inclusion in subsequent multivariate analyses; all continuous variables met criteria for normalcy according to these tests. Bivariate analyses (Wald chi-square tests) were conducted to determine the degree to which each predictor was related to diabetes status.

In multivariate analyses, categorical variables with more than two levels (e.g., “ethnicity” in NHANES) were entered into the model as ordinal variables. SAS PROC LOGISTIC (for ACES data) and PROC SURVEYLOGISTIC (for NHANES data, given the complex survey design) were used for logistic regression analysis. For the NHANES data, the logistic regression models were adjusted for the complex sampling design by using Mobile Examination Center (MEC) weights. Odds ratios and 95% Wald confidence limits were reported. Multivariate analyses statistically controlled for age, ethnicity, sex, education level, and body mass index.

Further analyses were conducted using the NHANES data, which included health behavior variables associated with diabetes that were not included in the ACES dataset, to gain a more robust understanding of the way health behaviors contributed to the relationship between depression and diabetes. These analyses allowed for evaluation of the association of depressive symptoms with diabetes after controlling for health behavior variables, which included: smoking (never = 0, former = 1, current = 2), sedentary behavior (self-reported lack of any physical activity; active = 0, sedentary = 1), and self-rated healthiness of diet (poor/fair = 1, good/very good/excellent = 0). This model also controlled for inflammation (C-reactive protein from laboratory blood draw, measured quantitatively).

The Institutional Review Board at the University of North Carolina at Chapel Hill reviewed the protocol for the present study and exempted it, as it involved only secondary analysis of de-identified data. Participants in each of the constituent studies provided informed consent at the time of data collection, and each parent study received approval and oversight from its respective ethics board.

## Results

### Sample characteristics

Descriptive statistics for the 3072 NHANES participants and the 2300 ACES participants are presented in Table 1. In both datasets, approximately one in five respondents had diabetes and 12–13% met the scale’s requirements for significant depressive symptomatology. The lifetime prevalence or “history” of depression in ACES was nearly 23%, whereas the prevalence of “recent” (12-month) depression was 8%. The ACES sample was slightly older, was more likely to be female, and had fewer Caucasians than the NHANES sample. The NHANES sample had more than twice as many respondents having pursued education beyond high school. The mean body mass indexes of both samples were comparable at 29 kg/m^2^. For the PHQ-9 (NHANES depressive symptoms measure), the raw Cronbach’s alpha was 0.85 in the present data, and raw alpha for the CES-D (ACES depressive symptoms measure) was 0.91 for the present data. Thus, the items in the scales were cohesive.

**Table 1:** Description and comparison of NHANES and ACES samples

Characteristic	NHANES (*n* = 3072)	ACES (*n* = 2300)
Percent with diabetes	19.2%	20.7%
Percent with depression or mean depressive symptoms score	PHQ-9: 3.2 out of 27, SE^a^ = 0.2	CES-D: 8.1 out of 60, SD^b^ = 9.1 Recent (12 months): 8.1% Lifetime: 22.9%
Mean age	59.5 years, SE = 0.3	65.1 years, SD = 10.5
Ethnicity	African American: 10.0% Latino: 8.7% Caucasian: 76.2% Other: 5.1%	African American: 33.8% Caucasian: 66.2%
Sex: Female	53.6%	68.1%
Education: High school or less	47.3%	75.0%
Mean body mass index	29.0, SE = 0.2	29.8, SD = 6.8

^a^SD = Standard Error

^b^SE = Standard Deviation

### Bivariate models

In NHANES, without adjusting for covariates, every 1-point increase in PHQ-9 score above the mean was associated with a 5% increase in odds of having diabetes [OR: 1.05 (95% CL: 1.03, 1.07)] (Table 2). In general, being older, being African American or Latino, having a high school education or less, and high BMI were each independently associated with increased diabetes risk over their respective comparison groups.

**Table 2:** Odds ratios and confidence intervals indicating bivariate associations with diabetes status

	NHANES		ACES	
	*n* = 3072		*n* = 2300	
Variable name	OR^a^	95% CI^b^	OR	95% CI
Depressive symptoms	**1.05**	**1.03, 1.07**	**1.02**	**1.01, 1.03**
Depression, recent	**–**	**–**	**1.58**	**1.13, 2.20**
Depression, lifetime	**–**	**–**	**1.35**	**1.07, 1.70**
Age (in years)	**1.03**	**1.02, 1.04**	**1.00**	**0.99, 1.01**
Ethnicity (reference: Caucasian)				
African American	**2.46**	**1.86, 3.26**	**1.63**	**1.32, 2.00**
Latino	**1.76**	**1.29, 2.39**	**–**	**–**
Other	1.14	0.56, 2.32	–	–
Sex: Female (reference: male)	0.84	0.69, 1.02	1.23	0.99, 1.53
Education (reference: more than high school)	**2.02**	**1.66, 2.46**	**1.65**	**1.30, 2.09**
Body mass index (in kg/m^2^)	**1.12**	**1.09, 1.15**	**1.07**	**1.06, 1.09**

Boldface indicates statistical significance (*p* < 0.05)

^a^OR = Odds Ratio

^b^CI = 95% Wald Confidence Interval

For the ACES data, before adjusting for covariates, depressive symptoms were independently associated with diabetes status such that a 1-point increase in CES-D score was associated with a 2% increase in likelihood of having diabetes [OR: 1.02 (1.01, 1.03)]. There was a 58% increase in diabetes risk for recent depression [OR: 1.58 (1.13, 2.20)] and 35% for lifetime depression [OR: 1.35 (1.07, 1.70)]. Being African American, having a high BMI, and having a high school education or less each increased the odds of diabetes. Age was not significantly associated with odds of having diabetes [OR: 1.00 (0.99, 1.01)], but it was included in the multivariate model for its theoretical and clinical importance.

### Multivariate models

#### NHANES

As shown in Table 3, the relationship between depressive symptoms and diabetes status seen in the bivariate model persisted after adjusting for sex, ethnicity, age, education, and BMI [OR: 1.05 (1.03, 1.07)]. Because depressive symptoms were measured continuously, the odds ratio indicates that the odds of having diabetes increased by 5% for every 1-point increase in PHQ-9 score above the mean.

**Table 3:** ORs and CIs for the multivariate and full NHANES models (*n* = 3072)

	Main Model		Main model + health behaviors + inflammation	
Variable name	OR^a^	95% CI^b^	OR	95% CI
Depressive symptoms (PHQ-9 score)	**1.05**	**1.03, 1.07**	**1.04**	**1.02, 1.06**
Age (in years)	**1.05**	**1.04, 1.06**	**1.05**	**1.04, 1.06**
Ethnicity (reference: Caucasian)				
African American	**2.45**	**1.85, 3.25**	**2.51**	**1.91, 3.30**
Latino	**1.78**	**1.32, 2.40**	**1.77**	**1.34, 2.33**
Other	1.79	0.95, 3.37	1.89	0.96, 3.70
Sex: Female (reference: male)	**0.66**	**0.51, 0.85**	**0.64**	**0.49, 0.85**
Education (reference: more than high school)	**1.68**	**1.29, 2.17**	**1.52**	**1.19, 1.94**
Body mass index (in kg/m^2^)	**1.13**	**1.10, 1.16**	**1.12**	**1.09, 1.16**
Smoking (reference: Never)				
Current	–	–	0.98	0.71, 1.36
Former	–	–	1.04	0.67, 1.61
Diet (reference: good/very good/excellent)	–	–	1.07	0.70, 1.63
Sedentariness (reference: active)	–	–	**1.58**	**1.23, 2.03**
C-reactive protein (in mg/L)	–	–	0.99	0.91, 1.07

Boldface indicates statistical significance (*p* < 0.05)

^a^OR = Odds Ratio

^b^CI = 95% Wald Confidence Interval

Odds ratios were significant for all covariates except the “other” level of ethnicity, indicating protective effects for being female, being Caucasian, being younger (closer to age 40), and having at least some postsecondary education. For every year of age over the mean, the odds of having diabetes increased by 5% [OR: 1.05 (CI: 1.04, 1.06)]. The odds of diabetes were 2.4 times as high for African Americans and 1.8 times as high for Latinos as for Caucasians. The odds of diabetes in women were about one-third lower than those for men [OR: 0.66 (CI: 0.51, 0.85)]. Having a high school diploma or less was associated with a 68% increase in odds for diabetes compared to those who have at least some post-secondary education [OR: 1.68 (CI: 1.29, 2.17)]. Every 1 kg/m^2^ increase in BMI over the mean was associated with a 13% higher odds of having diabetes [OR: 1.13 (CI: 1.10, 1.16)].

### NHANES plus behavioral variables and inflammation

The relationship between depressive symptoms and diabetes status persisted even after the addition to the multivariate model of the three health behavior variables (smoking, diet, and sedentary behavior) and inflammation. For every 1-point increase in PHQ-9 score over the mean, odds of diabetes rose by 4% [OR: 1.04 (CI: 1.02, 1.06)] (Table 3). Former smoking, current smoking, inflammation, and self-reported unhealthy diet were not significantly associated with diabetes status in the multivariate model. Being sedentary, however, was associated with a 58% increase in odds of having diabetes as compared to having at least some physical activity [OR: 1.58 (CI: 1.23, 2.03)].

### Aces

As shown in Table 4, self-reported diabetes was associated with CES-D depressive symptoms score [OR: 1.02 (CI: 1.01, 1.03)] and CIDI report of recent [OR: 1.49 (CI: 1.03, 2.13)] and lifetime [OR: 1.40 (CI: 1.09, 1.81)] depression after adjusting for covariates. Being older, having African American ethnicity, having a high school diploma or less, and having higher BMI were associated with increased odds of diabetes, while female gender was not significant in any model.

**Table 4:** ORs and CIs for the multivariate model in ACES (*n* = 2300)

	CES-D		Recent Depression		Lifetime Depression	
Variable name	OR^a^	95% CI^b^	OR	95% CI	OR	95% CI
Depression indicator	**1.02**	**1.01, 1.03**	**1.49**	**1.03, 2.13**	**1.40**	**1.09, 1.81**
Age (in years)	**1.02**	**1.01, 1.03**	**1.02**	**1.01, 1.03**	**1.02**	**1.01, 1.03**
African American ethnicity (reference: Caucasian)	**1.31**	**1.05, 1.63**	**1.34**	**1.08, 1.68**	**1.38**	**1.11, 1.73**
Sex: Female (reference: male)	1.02	0.81, 1.29	1.04	0.82, 1.30	1.01	0.80, 1.27
Education (reference: more than high school)	**1.35**	**1.05, 1.73**	**1.40**	**1.09, 1.79**	**1.43**	**1.12, 1.83**
Body mass index (in kg/m^2^)	**1.07**	**1.06, 1.09**	**1.07**	**1.06, 1.09**	**1.07**	**1.06, 1.09**

Boldface indicates statistical significance (p < 0.05)

^a^OR = Odds Ratio

^b^CI = 95% Wald Confidence Interval

## Discussion

This study demonstrated a consistent and robust relationship between depression and diabetes. These findings were substantial and significant across instruments used to measure depression (PHQ-9, CIDI, CES-D), timing of depression (recent versus lifetime exposure), classification of the “depression” variable (diagnosable depression versus depressive symptomatology), and study context (nationally representative versus representative of a specific rural county). The relationship between depression and diabetes persisted after controlling for the effects of body mass index and demographic characteristics on diabetes status and, for NHANES, remained robust even with the addition of three health behavior variables and a measure of inflammation.

The present findings add to empirical knowledge the observation that lifetime history of depression is associated with increased diabetes risk. Most previous studies of the relationship between depression and diabetes have used measures of depressive symptoms taken at the time of the baseline interview or within the previous year [22, 32, 33]. Having any history of experiencing depression was associated with significantly increased odds of having diabetes when compared to those who never had depression. The odds of diabetes diagnosis were sizeable for all measures of depression, and they remained robust across depression diagnosed proximally or over the lifetime and for both the nationally representative sample and the smaller rural study sample. Additional research is warranted to gain a better understanding of the relative importance of depression compared to other variables in the etiology of diabetes; some mechanisms, such as inflammation, [34] may impact both diabetes and depression. The findings here imply mental health, and specifically depression, should be considered when designing interventions.

Treatment studies suggest a causal association between diabetes and depression, although the direction of causality may be complex. When depression is treated in people with diagnosed diabetes, hemoglobin A_1_c (HbA_1_c), a biomarker for glucose control, decreases moderately [35]. The elevated risk for diabetes associated with depression remains even when other recognized diabetes risk factors are considered, including risk conferred by some antidepressants [32], poor diet, [36, 37] family history, [37] inflammation, [33] and sedentary lifestyle [36, 38].

### Limitations

As with many studies of depression and diabetes, this study uses mostly cross-sectional data, precluding inferences of causality. It remains unclear whether depression produces diabetes, is produced by it, or co-evolves with it. However, history of depression could hint at depression’s contribution to diabetes risk based on a retrospective-prospective study design [39]. Participants were asked to reflect retrospectively on symptoms they have experienced over the course of their lives, and these reflections were used by the CIDI computerized adaptive test to judge whether the symptoms were sufficient for a diagnosis of depression [28]. Then, lifetime depression status was used as a predictor of diabetes, providing some evidence for the claim that the two conditions are causally related.

There are other limitations to the present work. Researchers were unable to exclude individuals with type 1 diabetes from either analysis. For ACES, diabetes status was dichotomous and entirely self-reported, and no data were collected to indicate the type of diabetes a participant had. Also, it is no longer safe to assume that all diabetes that occurs during or before adolescence is Type 1, given the rise of Type 2 diabetes among teenagers [9]. However, it is estimated that only 5% of all diabetes cases are Type 1 [9]. Another limitation is that both datasets relied heavily on self-reported data for depression rather than clinical interviews or formal diagnoses, although the reliability and validity of instruments used is well-established [25–27, 29]. Self-report data are vulnerable to recall bias and other threats to validity. Finally, these analyses did not control for conditions that are frequently associated with depression and may also be associated with diabetes, such as anxiety and sleep disorders [5, 6].

Although generality cannot be proven, the present findings add substantially to previous evidence that the relationships between depression and diabetes are robust across methods and populations. The data analyzed were derived from large, community-based samples. The NHANES random sample purports to be representative of the United States population when properly weighted, so results using these data should be generalizable to individuals outside of those studied directly. The ACES dataset was a biracial, older-aged sample from a rural North Carolina county and therefore may be less generalizable than NHANES. Nevertheless, taken together, the findings from national and local data are informative.

## Conclusions

The strength of the relationship between lifetime experience of depression and diabetes risk suggests that interventions designed to lessen the burden of diabetes should focus on depression that occurs at any time over a person’s lifespan rather than only that which is detected immediately at the time of diabetes diagnosis or treatment. Much of the ongoing research in this field is biomedical, yet a multilevel public health model is needed to move this research forward by considering the myriad factors that contribute to each health outcome. This wider-lens model would also allow researchers and clinicians to include social determinants like education, which in the present analyses had a strong relationship with diabetes even after controlling for depression, in interventions.

The results from the present analyses demand the reconstruction of the current biomedical model to be more inclusive of psychosocial factors associated with disease. This model can then be used to develop interventions that will treat both body and mind. For example, several randomized controlled trials are underway in which researchers are using cognitive behavioral therapy to relieve depression among people living with diabetes, with the ultimate goal of managing their diabetes more effectively [40, 41].

Amidst the burgeoning literature addressing relationships among depression and other mental health indicators on the one hand, and diabetes and other physical health conditions on the other, the present findings add evidence of the robustness of the depression-diabetes connection across several populations as well as sampling, measurement, and analytic approaches. They also add the novel finding that a reported history of depressive symptoms is predictive of later diabetes. In addition to reinforcing the general interest in these issues, the present findings add evidence for the importance of depression as an indicator for both prevention and treatment of diabetes.

## Abbreviations

ACES: Arthritis, Coping, and Emotion Study
CDC: Centers for Disease Control and Prevention
CES-D: Center for Epidemiologic Studies Depression inventory
CIDI: Composite International Diagnostic Interview
DSM-III-R: Third edition of *Diagnostic and Statistical Manual for Mental Disorders*, Revised
DSM-IV: Fourth edition of *Diagnostic and Statistical Manual for Mental Disorders*
FPG: Fasting plasma glucose
HbA1c: Glycated hemoglobin (hemoglobin A1c)
JoCo OA: Johnston County Osteoarthritis Study
MEC: NHANES Mobile Examination Center
NC: North Carolina
NHANES: National Health And Nutrition Examination Survey
PHQ-9: 9-Item Patient Health Questionnaire
US: United States of America.
